# Single-session alcohol sclerotherapy of symptomatic liver cysts using 10–20 min of ethanol exposure: no recurrence at 2–16 years of follow-up

**DOI:** 10.1007/s00261-016-0769-9

**Published:** 2016-05-07

**Authors:** Trond Bjerke Larssen, Asgaut Viste, Arild Horn, Ingfrid Salvesen Haldorsen, Ansgar Espeland

**Affiliations:** 1 Department of Radiology, Haukeland University Hospital, Jonas Liesvei 65, 5021 Bergen, Norway; 2 Department of Clinical Medicine, University of Bergen, Pb 7804, 5020 Bergen, Norway; 3 Department of Acute and Gastrointestinal Surgery, Haukeland University Hospital, Jonas Liesvei 65, 5021 Bergen, Norway

**Keywords:** Cysts, Ethanol, Liver diseases, Sclerotherapy, Treatment outcome

## Abstract

**Purpose:**

To assess long-term results after single-session alcohol sclerotherapy of symptomatic benign liver cysts performed with maximum 20 min of exposure to alcohol.

**Methods:**

We included 47 patients aged 32–88 years (42 women, 5 men) with 51 benign non-parasitic liver cysts that were exposed to ethanol for 7–20 min in a single sclerotherapy session and were followed for at least 24 months. Each cyst was emptied before injecting ethanol (10% of cyst volume, but maximum 100 mL) into it. The patient rotated from side to side to facilitate contact between ethanol and the whole cyst wall. Pre-treatment cyst volume was defined as the volume of aspirated cyst fluid after complete emptying of the cyst. Follow-up cyst volume was estimated based on computed tomography images.

**Results:**

Cyst volumes were 30–4900 (median 520) mL at pre-treatment and 0–230 (median 1) mL at 24–193 (median 56) months follow-up, a reduction of 83–100% (median 99.7%). No cyst required repeated treatment during the follow-up. Median volume reduction was 99.7% at median 49 months of follow-up for 35 cysts exposed to ethanol for 7–10 min vs. 99.6% at median 75 months of follow-up for 16 cysts exposed for 20 min (*p* = 0.83, Mann–Whitney test). Ethanol intoxication occurred in one patient. There were no other complications except for pain.

**Conclusion:**

Long-term results of single-session alcohol sclerotherapy performed with maximum 20 min of exposure to ethanol were satisfactory with no sign of recurrence of cyst fluid.

Alcohol sclerotherapy may be an effective treatment for solitary symptomatic liver cysts and for dominant symptomatic cysts in polycystic livers [1–23]. However, there is much variation in the time the cyst is exposed to alcohol during the procedure (10–240 min), the volume of alcohol applied, and in the use of one single [5–9, 13, 16, 21, 22] or multiple [3, 4, 23] sclerotherapy sessions. These factors affect the laboratory time needed, the risk of alcohol intoxication, and the procedural costs. Thus, research has been recommended to standardize and simplify the method of sclerotherapy [24, 25]. Further studies are also needed on long-term results of sclerotherapy of liver cysts [9, 22, 23]. Earlier reports by Larssen et al. suggest good results of exposing liver cysts to alcohol for 10 or 20 min in a single sclerotherapy session [5, 8, 9]. Here we report results of such a treatment in a larger material after a minimum of two-year follow-up. The purpose of the present study was to assess long-term results after single-session alcohol sclerotherapy of symptomatic benign liver cysts performed with maximum 20 min of exposure to alcohol.

## Materials and methods

This study included 47 patients with at least 24 months of follow-up after alcohol sclerotherapy of symptomatic benign liver cysts performed at our hospital from 1993 to 2010 according to the procedure described below. Patient data were recorded prospectively. Among 68 consecutive patients treated with alcohol sclerotherapy for solitary symptomatic liver cysts or for dominant symptomatic cysts in polycystic livers, we excluded 17 patients followed for <24 months and 4 patients lost to follow-up. The follow-up protocol initially included imaging to assess cyst volume within 2 months, and at 6, 12, and 24 months. We later reduced follow-up imaging during the first year, since it typically showed temporary recollection of cyst fluid that did not require treatment (see “Results” section). The material does not include symptomatic polycystic livers containing only innumerable small cysts. Gastrointestinal surgeons at the hospital with extensive experience in evaluating liver disease had evaluated all patients and judged them in need of invasive treatment due to symptoms from a liver cyst, e.g., pain, fullness, early satiety. Since 1993 at our institution, nearly all such patients have undergone alcohol sclerotherapy rather than primary surgery. If the cause of symptoms was uncertain, the cyst was emptied to test if the symptoms vanished. We did not perform alcohol sclerotherapy of hydatid cysts, neoplastic cysts, or cyst-like ecstasies of the intrahepatic biliary ducts. Contraindications to sclerotherapy were coagulopathy, communication between the cyst and the biliary tree or the peritoneal cavity, and failure to aspirate injected contrast medium from the cyst.

### Sclerotherapy procedure

Premedication (50–100 mg pethidine, 0.6 mg atropine) was used from 1993 to 2002. Since 2002, no premedication was used, but an anesthetic nurse was always present during the procedure and provided the sedation and analgesics needed (midazolam, alfentanil).

The patient was positioned supine on an angiographic table. The cyst was punctured under ultrasonographic guidance, usually with a 1.2-mm-diameter 20-cm-long needle. If possible, the needle was inserted through 1-3 cm of liver tissue to prevent leakage from the cyst. A stiff guide wire was introduced and a 30-cm 7 French (2.3 mm outer diameter) pigtail catheter (PBN Medicals, Denmark; Argon Medical Devices, Denmark) was pushed as far as possible into the cyst and fixed to the skin by adhesive tapes. After complete emptying of the cyst, contrast material was injected and radiographs taken to confirm the position of the catheter and to exclude communication with the biliary ducts and leakage of contrast into the peritoneal cavity. If this occurred, or aspiration of contrast material from the cyst failed, the procedure was discontinued. Otherwise, ethanol was injected in an amount of 10% of the cyst volume, but never more than 100 mL. To avoid dilution of ethanol, great care was taken to empty the cyst completely before injecting ethanol into it. To achieve satisfactory contact between ethanol and cyst wall, it was routine to change the patient position from prone to supine and right to left lateral decubitus position at least two times during the procedure. We had tested the pigtail catheter for use with alcohol ex vivo. The manufacturer (Argon Medical Devices) has tested the catheter in contact with 100% alcohol and found no sign of degradation or leakage up to 6 weeks (Argon Report REP1-137707, Nov 07, 2013). Alcohol was injected using regular plastic syringes that were usually filled with alcohol within 3 min from the injection time.

Cysts were exposed to ethanol for 20 min (from 1993 to 1995) or 10 min (from December 1995 to June 1999). From June 1999, cysts smaller than 1000 mL were exposed to ethanol once for 10 min, whereas cysts with volumes of at least 1000 mL were exposed to ethanol twice in a single session, each time with 100 mL of ethanol for 10 min. We used 96% ethanol during the period 1993–1999 and 99% ethanol since July 1999.

Finally, all alcohol was evacuated, the cyst was irrigated with saline (to avoid leakage of ethanol during withdrawal of the catheter), and the catheter was removed. The patient rested in bed for 4 h and if asymptomatic was discharged from the hospital the same afternoon. The patients were observed for any signs of alcohol intoxication during and after the procedure. The blood alcohol level was not measured routinely, since it was ≤0.3 mg/g in initial patients.

### Cyst volume

Pre-treatment cyst volume was estimated as the volume aspirated on the date of sclerotherapy when emptying the cyst. At follow-up, one experienced radiologist measured the three largest perpendicular cyst diameters d1, d2, and d3 on computed tomography (CT) images, and calculated cyst volume using the formula of an ellipsoid: volume = d1 × d2 × d3 × 0.523 [26].

### Statistical analysis

WINPEPI version 11.60 (http://www.brixtonhealth.com/pepi4windows.html) was applied for statistical analysis. The Mann–Whitney test for comparing medians was used to compare pre-treatment cyst volumes, follow-up cyst volumes, reduction of cyst volumes at follow-up, and follow-up times between cysts exposed to alcohol for 10 min and cysts exposed to alcohol for 20 min. All *p* values are two-tailed. *p* < 0.05 indicated statistical significance.

## Results

Forty-seven patients (42 women and five men) aged 32–88 (median 61) years had 51 liver cysts exposed to ethanol for 7–20 (median 10) minutes and were followed for 24–193 (median 56) months. Six cysts in five patients were in polycystic livers and 45 cysts in 42 patients were not.

In the total sample, median cyst volumes were 520 mL at pre-treatment and 1 mL—or median 99.7% smaller—at the last follow-up (Table 1). None of the 51 treated cysts required repeated treatment during the reported follow-up period due to insufficient effect of the sclerotherapy.

**Table 1:** Single-session alcohol sclerotherapy of 51 symptomatic benign liver cysts in 47 patients

	Age (years)	Women/men (numbers)	Original cyst volume (mL)	Follow-up cyst volume (mL)	Reduction of cyst volume at follow-up (%)	Time of follow-up (months)
Total sample	61 (32–88)	42/5	520 (30–4900)	1 (0–230)	99.7 (83–100)	56 (24–193)
Alcohol exposure						
10 min^a^ (35 cysts, 32 patients^b^)	61 (43–88)	27/5	394 (30–4110)	1 (0–230)	99.7 (83–100)	49 (24–193)
20 min (16 cysts, 16 patients^b^)	68 (32–86)	15/1	1525 (200–4900)	5 (0–188)	99.6 (91–100)	75 (24–171)

Values are median (range) except values for women/men

^a^Includes two cysts exposed for 7 and 8 min, respectively

^b^One patient (a man)is included in both alcohol exposure groups because he had one cyst exposed to alcohol for 10 min and another cyst exposed to alcohol for 20 min

Median volume reduction was 99.7% for 35 cysts exposed to ethanol for 7–10 min vs. 99.6% for 16 cysts exposed to alcohol for 20 min (*p* = 0.83, Table 1). Cysts exposed for 7–10 min vs. 20 min did not differ significantly in volume at follow-up (*p* = 0.28) or time to follow up (*p* = 0.24) (Table 1), but were originally smaller (median 394 vs. 1525 mL, *p* = 0.004; Table 1). This was expected, since cysts ≥1000 mL were exposed to alcohol for 20 min from 1999. Seven (of 18) cysts ≥1000 mL were exposed to alcohol for 7–10 min, and were 88–100% (median 99%) smaller at 78–193 months (median 11 years) of follow-up.

We followed 11 cysts in 10 patients for more than 10 years. These cysts were 88–100% (median 100%) smaller at 127–193 months (median 12 years) of follow-up.

**Fig. 1:**
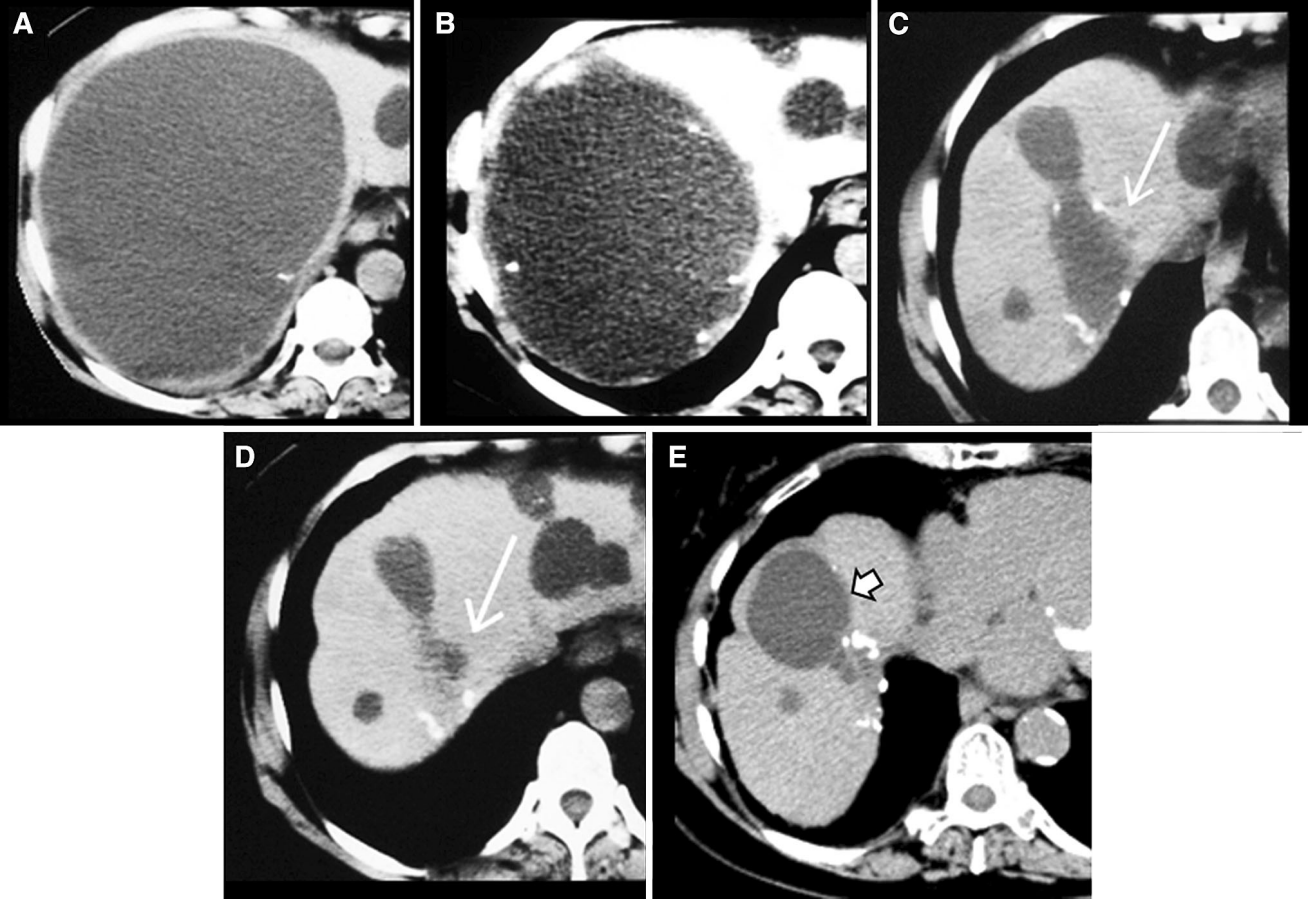
**A** 1750 mL liver cyst in a 71-year-old woman before sclerotherapy (**A**). Recollection of 850 mL cyst fluid was observed 24 days after sclerotherapy (**B**). However, the cyst (*long white arrows*) was smaller at 6 months without repeated sclerotherapy (**C**), had shrank to 99% of its pre-treatment size at 17 months (**D**), and showed no signs of recurrence at 193 months post treatment (**E**). An adjacent cyst (*short white arrow*) had grown slowly to 65 mL at 193 months post treatment (**E**)

CT performed within three months after sclerotherapy of 17 cysts in 16 patients showed recollection of cyst fluid in all. The fluid later diminished in all patients without therapy (Fig. 1). At the first follow-up at 0.3–3 (median 1) months, the 17 cysts measured 9–100% (median 39%) of their original volume of 30–4900 (median 1250) mL. All 17 cysts then shrank during the first year. Cyst volume was 0–30% (median 5%) of the original volume at 4.5–6 months (data on 13 cysts) and 0–36% (median 2%) at 10–14 months (data on 13 partly different cysts). At the last follow-up, the 17 cysts had shrank to 0–12% (median 0.3%) of their initial volume.

There was one major procedure related complication: ethanol intoxication in a 77-year-old woman following exposure of one 4900 mL cyst to 100 mL alcohol for 10 min twice in one session (respiratory failure, alcohol smell from breath, blood alcohol concentration 1.95 mg/g; morphine antidote given, uneventful recovery; cyst disappeared at 77 months of follow-up). A minor complication was abdominal pain during the procedure. This was no longer a problem after we abandoned premedication and started to use analgesics according to individual needs and to irrigate the cysts with saline before removing the catheter, preventing remnants of ethanol within it to cause pain during removal.

## Discussion

In this study, liver cysts exposed to alcohol for a maximum of 20 min in one treatment session were 83–100% (median 97.7%) smaller at 2–16 years of follow-up. No cyst required repeated treatment during the follow-up. Even for large cysts, we used maximum 100 mL of alcohol once or twice in a single session. The procedure caused alcohol intoxication in one patient (2%) with an uneventful recovery. The brief exposure of liver cysts to a relatively small amount of alcohol was thus safe and prevented recurrence of cyst fluid in the long term.

Alcohol should be in contact with the whole cyst cavity during the treatment in order to fix the epithelial cells and disable their ability to secret fluid [1, 15, 21, 27]. Alcohol can also induce a temporary inflammatory reaction in a cystic wall deprived of vital epithelium [28]. This may explain the transitory re-accumulation of cyst fluid seen in the present and other studies [5, 6]. Importantly, cyst fluid re-accumulated within three months but diminished without treatment.

Some parts of our procedure may be essential when exposing liver cysts to limited amounts of alcohol for a short time. First, in order to avoid dilution of alcohol concentration, it is important to empty the cyst completely before injecting alcohol into it. Second, after alcohol injection, the patient should rotate from side to side to facilitate contact between alcohol and every part of the cyst, especially when the cyst is large and may have a multifolded wall when emptied. Third, to make it easier for the patient to co-operate and change position, we advise effective pain management as needed during the procedure rather than general premedication.

We found good results of exposing liver cysts to alcohol for only 10 min, based on treatment of 35 cysts. Larssen et al. have previously reported promising results of exposing liver cysts to ethanol for 10 min in a study comprising only 10 cysts [8]. The present findings suggest that an alcohol exposure time of 10 min may be equally effective as an alcohol exposure time of 20 min in many patients. An alcohol exposure time of 10 min may be effective also for cysts >1000 mL, but was used in only seven such cysts in our study. In their recent retrospective study, Akhan et al. reported successful results of exposing 39 liver cysts to alcohol for 10 min, but all cysts in their material were <700 mL [22]. Therefore, the effectiveness of exposing cysts >1000 mL to alcohol for 10 min needs further validation.

Provided the sclerotherapy remains effective, briefer exposure to alcohol during the procedure may be advantageous by reducing alcohol intoxication risk, interventional laboratory time, and duration of hospitalization. In studies of liver cysts exposed to alcohol for 60 min [2, 6], all patients had elevated alcohol levels in the blood, and both prolonged exposure and increased volume of alcohol increased the risk of alcohol intoxication.

Our study had strengths and limitations. It represented day-to-day radiological work, making the results highly relevant to clinical practice. Patients and data were noted prospectively, avoiding retrospective identification of patients in medical records. The follow-up period was longer and the material slightly larger (24–193 months, median 56 months; 51 cysts) than in recent studies by Akhan et al. [22] (4–173 months, median 38 months; 39 cysts) and Jang et al. [23] (12–106 months, mean 33 months; 43 cysts). We used CT and the same observer (not ultrasonography performed by different observers) to estimate cyst volumes at follow-up by means of a validated method for estimating organ volumes [26, 29]. However, we did not report on course of symptoms, except that no cyst required repeated treatment. Since prior research had shown good effect of reduced cyst volume on symptoms [2, 5–8], we rather focused on whether our treatment procedure ensured small cyst volumes in the long term. As part of the daily clinical practice, we adjusted the procedure slightly during the study period based on accumulated experience. We reduced the alcohol exposure time from 20 to 10 min based on good results in three initial patients in whom the procedure had to be discontinued due to pain. We later exposed cysts >1000 mL for 20 min (10 min twice) to be more certain of effect. However, our study design was not optimal for comparing results between 10 and 20 min of alcohol exposure, which would have required a randomized trial.

*In conclusion*, the employed procedure of alcohol sclerotherapy of liver cysts varies considerably between institutions. This study showed that there is a durable response to sclerotherapy performed with 10–20 min of exposure to ethanol in a single session.
